# Oxylipins in Atherosclerosis: Their Role in Inflammation, Diagnosis, and Therapeutic Perspectives

**DOI:** 10.3390/ijms262110577

**Published:** 2025-10-30

**Authors:** Dmitry V. Chistyakov, Vasiliy V. Chistyakov, Marina G. Sergeeva

**Affiliations:** 1 Belozersky Institute of Physico-Chemical Biology, Lomonosov Moscow State University, 119992 Moscow, Russia; 2 Institute of Pharmacy and Biotechnology (IPhB), Peoples’ Friendship University of Russia (RUDN University), 117198 Moscow, Russia; 3 Faculty of Bioengineering and Bioinformatics, Lomonosov Moscow State University, 119234 Moscow, Russia

**Keywords:** atherosclerosis, inflammation, oxylipins, biomarkers, lipidomics, cardiovascular disease

## Abstract

Atherosclerosis, the principal pathology underlying cardiovascular diseases, is now recognized as a chronic inflammatory disorder of the arterial wall. This review focuses on the central role of oxylipins, a diverse family of bioactive lipids derived from polyunsaturated fatty acids (PUFAs), in the inflammatory processes driving atherosclerosis. We synthesize evidence that oxylipins produced via cyclooxygenase (COX), lipoxygenase (LOX), cytochrome P450 (CYP), anandamide (AEA) pathways and non-enzymatic transformations of PUFAs are pivotal modulators of vascular function, immune cell recruitment, and plaque stability. The balance between pro-inflammatory mediators and specialized pro-resolving mediators (SPMs) is critical; a shift towards inflammation underlies disease progression. Advances in lipidomics now enable comprehensive oxylipin profiling, revealing distinct signatures with significant diagnostic and prognostic potential for assessing coronary artery disease severity and predicting future cardiovascular events. Therapeutically, while current anti-inflammatory strategies target downstream pathways, this review highlights emerging approaches that modulate the oxylipin system directly. These include promoting SPMs synthesis through omega-3 supplementation, inhibiting pro-inflammatory leukotriene production, and preserving cardioprotective epoxyeicosatrienoic acids (EETs) via soluble epoxide hydrolase (sEH) inhibition. A deeper understanding of these complex oxylipin networks promises to yield novel biomarkers and targeted therapies designed to restore inflammatory homeostasis and combat atherosclerotic cardiovascular disease.

## 1. Introduction

Cardiovascular diseases (CVDs) remain the leading cause of mortality worldwide, with atherosclerosis as the primary underlying pathology. Atherosclerosis is now widely recognized as a chronic inflammatory disease of the arterial wall. Inflammation is a key mechanism that mediates the transformation of traditional risk factors into morphological changes and clinical symptoms [1,2,3]. The inflammatory response is a normal function of the innate immune system, characterized by a complex interplay of signaling molecules, particularly the release of cytokines and a diverse class of bioactive lipids known as oxylipins [4]. Pathologies arise when this complex interplay is disrupted. Oxylipins, derived from the enzymatic and non-enzymatic oxidation of polyunsaturated fatty acids (PUFAs), represent a diverse family of bioactive molecules that modulate inflammation, vascular function, and tissue repair [5,6,7]. The role of oxylipins, primarily prostaglandins and leukotrienes, in inflammatory processes and relative pathologies has been comprehensively analyzed in several notable reviews and research articles [8,9,10,11,12,13,14]. Detailed data on the pro- and anti-inflammatory functions of individual oxylipins can be found in review articles [15,16,17,18].

In recent decades, methods for comprehensive oxylipin profiling have emerged, significantly expanding our understanding of their role in various inflammatory diseases, including atherosclerosis. The ability to detect more than a hundred oxylipins simultaneously has expanded our understanding of their dual role as a crucial part of the inflammatory response [19]. This progress is paving the way for new therapeutic strategies to prevent and diagnose abnormal inflammatory responses. In this review, we analyze recent data indicating that alterations in oxylipin metabolism are an integral part of the onset and development of atherosclerosis. All cell types involved in atherosclerosis are capable of synthesizing oxylipins through various metabolic pathways and responding to their signals via specific receptors. Modern clinical research is increasingly focused on controlling inflammation, including methods for its suppression and the stimulation of natural resolution. Although the systemic functions of oxylipins in inflammation are not fully understood, analyzing their profiles deepens our understanding of atherosclerosis and opens new possibilities for mitigating its impact.

## 2. Pathogenesis of Atherosclerosis: A Cellular Perspective

The development of atherosclerotic lesions is a multi-stage process involving various cell types. A key role belongs to the interaction of endothelial cells, monocytes/macrophages, foam cells, smooth muscle cells, and platelets. Importantly, all these cells contribute to inflammatory processes by releasing cytokines and oxylipins [3,7,17,20]. Dysregulation of inflammatory crosstalk between these cells may underlie disease progression and its clinical complications.

The involvement of lipids in atherosclerosis has been studied for over a century (see the history of lipid research in the review by [21]). Initially, it was discovered that atherosclerotic plaques contain cholesterol; later, it was shown that a high-cholesterol diet causes atherosclerosis in rabbits [21]. Epidemiological studies subsequently confirmed the link between high blood cholesterol levels and the risk of cardiovascular diseases. In the second half of the 20th century, researchers focused on low-density lipoproteins (LDL) as the main carriers of cholesterol in the blood. It was established that LDL receptors in the liver regulate the levels of these lipoproteins. Mutations that reduce receptor function lead to elevated LDL levels and early coronary heart disease, while mutations that increase receptor activity lower LDL levels and disease risk [21]. Atherosclerosis was thus classified as a cholesterol storage disease, with elevated plasma cholesterol and LDL levels linked to its development [22,23,24]. These discoveries paved the way for statins—drugs that lower cholesterol by inhibiting the key enzyme in cholesterol synthesis, HMG-CoA reductase.

However, despite effective cholesterol reduction with statins, the residual inflammatory risk remains a serious unsolved problem [25]. This has stimulated the search for new therapeutic strategies focused on managing inflammation. The FDA recently approved low-dose colchicine (0.5 mg per day) as an anti-inflammatory therapy to be used in addition to statins to reduce the risk of myocardial infarction, stroke, coronary revascularization, and cardiovascular death [26]. Canakinumab (an anti-IL-1β antibody) demonstrated the benefit of targeting inflammation in chronic atherosclerosis [27]. Its use in the CANTOS trial was the first clinical study to show that anti-inflammatory treatment can reduce cardiovascular complications [28]. Currently, drugs inhibiting the pro-inflammatory interleukin-6 (IL-6), such as Ziltivekimab and Clazakizumab, are undergoing clinical trials for atherosclerosis therapy (see review [29]). Sodium-glucose cotransporter-2 (SGLT2) inhibitors also demonstrate anti-inflammatory potential [30]. Blockade of the IL-6 receptor, CC chemokine receptor 2, and CD20 are considered promising targets [30]. Although our understanding of the inflammatory response is still incomplete, the development of anti-inflammatory therapy represents a new opportunity for treating cardiovascular diseases [3,27].

It should be emphasized that modern anti-inflammatory therapy often aims to treat an already dysregulated inflammatory response. The trend of shifting research focus towards early diagnosis and attempts to slow disease progression, or even prevent its occurrence, has drawn attention to the development of diagnostic approaches and the study of processes that enhance the resolution of inflammatory responses [31]. This, in turn, has highlighted oxylipin metabolism, as many oxylipins function as pro-resolving mediators [32,33].

## 3. Biosynthesis of Oxylipins: Metabolic Pathways and Key Enzymes

**Figure 1:**
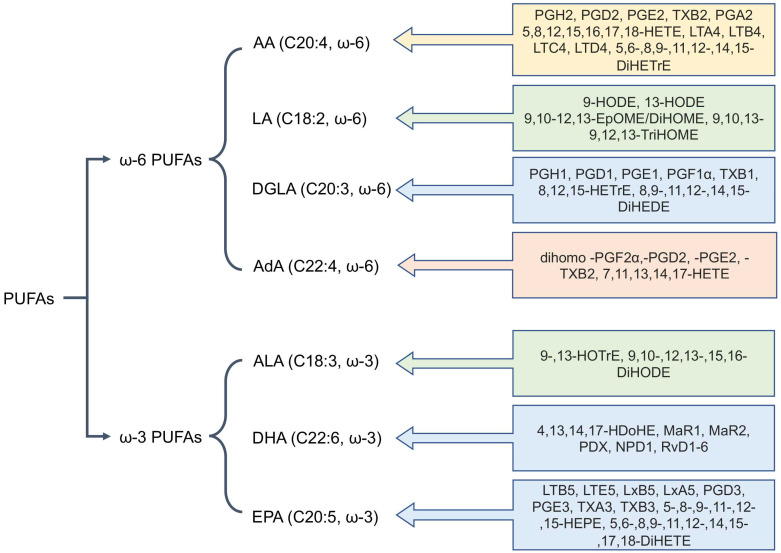
The main polyunsaturated fatty acids (PUFAs) involved in mammalian oxylipin metabolism. Abbreviations: Metabolites: AA—Arachidonic acid, LA—linoleic acid, DGLA—dihomo-γ-linolenic acid, AdA—adrenic acid, ALA—α-linolenic acid, EPA—eicosapentaenoic acid, DHA—docosahexaenoic acid.

Oxylipins are derivatives of polyunsaturated fatty acids (PUFAs) that possess a wide range of functions, participating in processes such as cell proliferation, apoptosis, blood coagulation, blood pressure regulation, and immune response [4,5,7,17]. Oxylipins are formed from various PUFAs, which are categorized based on the position of their double bonds into ω-6 and ω-3 families [4,5]. The most common PUFAs in humans are the ω-6 acids: arachidonic (AA, 20:4), linoleic (LA, 18:2), dihomo-γ-linolenic (DGLA, 20:3), and adrenic (AdA, 22:4) acids, as well as the ω-3 acids: α-linolenic (ALA, 18:3), eicosapentaenoic (EPA, 20:5), and docosahexaenoic (DHA, 22:6) acids [4,5]. Figure 1 shows the major PUFAs and their key metabolites.

The benefits of consuming ω-3 fatty acids over ω-6 fatty acids in the context of cardiovascular diseases have long been debated [34,35,36]. Potential interactions between statins and omega-3 fatty acids relevant for the prevention and treatment of atherosclerotic cardiovascular disease are discussed in the review by [37]. The role of dietary PUFAs and their impact on oxylipin synthesis is detailed in a recent review [17]. ω-3 fatty acids (EPA and DHA) compete with ω-6 AA and serve as a substrate for the synthesis of anti-inflammatory oxylipins, such as specialized pro-resolving mediators (SPMs), which promote the resolution of inflammation [38,39,40,41]. However, clinical data are conflicting: the JELIS [42,43] and REDUCE-IT [44] trials using high doses of pure EPA demonstrated a significant reduction in cardiovascular events. The RESPECT-EPA trial showed a trend towards benefit [45]. In contrast, studies of EPA/DHA mixtures (STRENGTH, VITAL, ASCEND) did not reveal significant benefits [46,47]. This difference is attributed to the unique effects of EPA and the stability of the formulations [35,42]. Omega-3 fatty acids have been shown to stabilize plaques by reducing their volume [48,49]. The Omega-3 Index is a biomarker of cardiovascular risk PMID: 27357102. A promising approach is to tailor therapy based on the Omega-3 Index and inflammatory biomarkers [35,36,50].

Understanding the mechanisms of PUFA and oxylipin involvement requires consideration of their biochemical metabolic and signaling pathways. PUFAs are mainly esterified at the sn-2 position of phospholipids and are released by the action of phospholipase A2 (PLA2) [7]. The PLA2 family includes more than 20 members, which generally lack strict specificity regarding the number of double bonds in PUFAs. The released PUFAs undergo further metabolism through five main pathways. Three are designated according to their key enzymes: the cyclooxygenase (COX), lipoxygenase (LOX), and cytochrome P450 monooxygenase (CYP450) pathways [5,51,52]. The anandamide (AEA) pathway and non-enzymatic transformations of PUFAs are also significant sources of oxylipins [53,54].

### 3.1. The Cyclooxygenase (COX) Pathway

**Figure 2:**
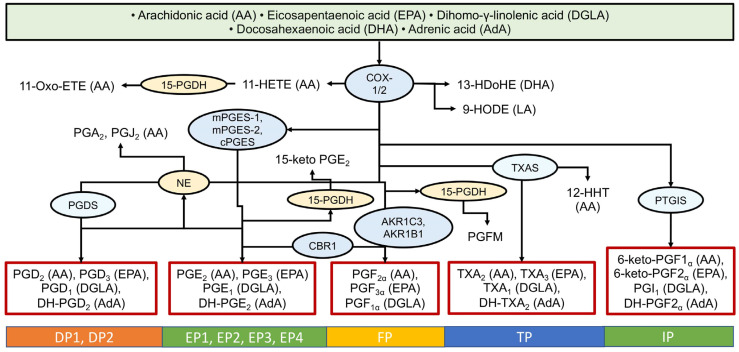
Biosynthesis of oxylipins via the cyclooxygenase (COX) pathway. The ovals show the main enzymes of oxylipin biosynthesis, and the precursor acids are indicated in brackets. Abbreviations: Metabolites: PGs—prostaglandins, TXs—thromboxanes; Proteins: COX-2—cyclooxygenase-2, mPGES—microsomal prostaglandin E synthases, PGDS—prostaglandin-D synthase, NE—non-enzymatically, CBR1—carbonyl reductase 1, AKRs—aldo-keto reductases, 15-PGDH—15-hydroxyprostaglandin dehydrogenase, PTGIS—Prostaglandin-I synthase; Receptors: DPs—prostaglandin D2 receptors, EPs—prostaglandin E receptors, FP—prostaglandin F receptor, TP—thromboxane receptor, PI—prostacyclin receptor.

The COX pathway involves the enzymes COX-1 and COX-2, which convert AA into prostaglandin H2 (PGH2), the precursor for prostaglandins of the E and D series, thromboxane’s (TX), and prostacyclin’s. These are formed through branching poly-enzymatic cascades (Figure 2). COX-1 is constitutively expressed and considered a housekeeping enzyme, while COX-2 is inducible and associated with inflammation [55]. The COX pathway converts arachidonic acid into pro-inflammatory prostaglandins (PGE2, PGD2, PGF2α), prostacyclin, and thromboxane A2 (TXA2), which is rapidly hydrolyzed to TXB2 [4,5]. However, this pathway also produces oxylipins from other PUFAs (Figure 2). There are nine main G-protein coupled receptors for COX-derived metabolites, named for their primary ligands: the PGD2 receptors (DP1, DP2), the PGE2 receptors (EP1-EP4), the PGF receptor (FP), the thromboxane receptor (TP), and the prostacyclin receptor (IP) [4,7].

Eicosanoids (derivatives of C20 fatty acids like AA and EPA) have long been studied in atherosclerosis (see, for example, [56]). Prostaglandins exhibit both pro- and anti-inflammatory effects [4,7]. Prostaglandins PGD2 and PGE2 readily undergo dehydration in vivo and in vitro, forming cyclopentenone prostaglandins (cyPG) of the J2 and A2 series [57,58]. While classic prostaglandins act through G protein-coupled receptors, cyPGs are actively transported into cells and interact with numerous intracellular targets, including signaling molecules and nuclear receptors. They exert anti-inflammatory effects and play a role in the resolution of inflammation [57,58,59]. Thus, during an inflammatory response, a shift from a pro-inflammatory signal to inflammation-resolving signals is observed. In addition to the context-dependent synthesis of oxylipins with divergent actions, the time-dependent change in the ratios of individual metabolites is also important.

The enzymes of the cyclooxygenase pathway are the targets of well-known anti-inflammatory drugs; therefore, these drugs are actively being tested in cardiovascular pathologies. It has been shown that COX-2 inhibitors increase cardiovascular risk due to an imbalance in the prostacyclin/thromboxane ratio [60]. Although the PRECISION trial demonstrated the cardiovascular safety of celecoxib [61], current guidelines contraindicate celecoxib in patients with established ischemic heart disease, peripheral arterial disease, cerebrovascular disease, or congestive heart failure [60,62]. It is noted that aspirin is effective for the secondary prevention of CVD [63], but its role in primary prevention is limited by the risk of bleeding [64]. Licofelone, a dual COX/5-LOX inhibitor, demonstrated preclinical efficacy in a rabbit model of atherosclerosis [65] but was not approved for clinical use.

It is well-known that the properties of prostaglandins change depending on the precursor fatty acid. For example, EPA-derived prostaglandins of series 3 can also bind to EP receptors but possess anti-inflammatory properties, unlike series 2 prostaglandins derived from AA [66]. This ability of omega-3 fatty acid metabolism to shift towards anti-inflammatory compounds may explain the positive correlation between diets high in omega-3 acids and slowed atherosclerosis progression, as repeatedly described (see, for example, the review [37]).

### 3.2. The Lipoxygenase (LOX) Pathway

**Figure 3:**
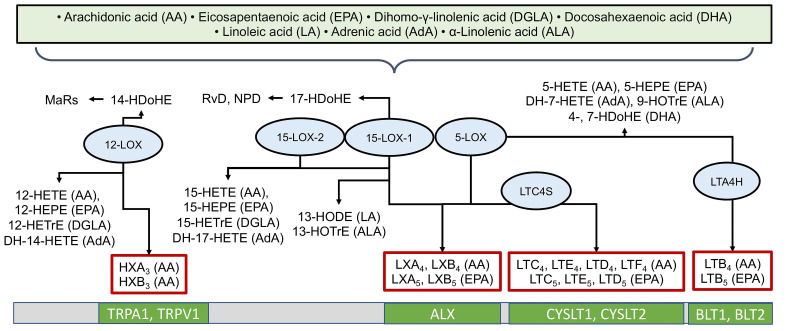
Biosynthesis of oxylipins via the lipoxygenase (LOX) pathway. The ovals show the main enzymes of oxylipin biosynthesis, and the precursor acids are indicated in brackets. Abbreviations: Metabolites: MaRs—maresins, HXs—hepoxilins, LTs—leukotrienes, LXs—lipoxins, HODEs—hydroxyoctadecadienoic acids, HDoHEs—hydroxydocosahexaenoic acids, HOTrE—hydroxyoctadecatrienoic acids, HETE—hydroxyeicosatetraenoic acids, HEPE—hydroxyeicosapentaenoic acids; Proteins: LOXs—lipoxygenases; Receptors: LTC4S—Leukotriene C4 synthase, LTA4H—Leukotriene-A4 hydrolase, TRPV1 and TRPA1—TRP channels, ALX—lipoxin receptor, CYSLTs—Cysteinyl-leukotriene receptors, BLTs—leukotriene B4 receptors.

The LOX pathway involves key enzymes such as 5-LOX, 12-LOX, and 15-LOX [67] in the formation of hydroxyeicosatetraenoic acids (HETEs), leukotrienes (LTs), lipoxins (LXs), and other physiologically active compounds [5,67]. LOX enzymes possess positional specificity; for example, when acting on arachidonic acid, 5-LOX generates 5-HETE and leukotrienes, 12-LOX generates 12-HETE, and 15-LOX generates 15-HETE and lipoxins [5,67]. The LOX pathway converts AA into pro-inflammatory leukotrienes (LTB4, LTC4, LTD4, LTE4) and anti-inflammatory lipoxins (LXA4, LXB4) [32,33]. LOX enzymes convert various PUFAs into a wide spectrum of compounds, including groups such as leukotrienes, resolvins (Rv), lipoxins (LX), maresins (MaR), hepoxilins (HX), as well as hydroxyoctadecadienoic (HODE), hydroxydocosahexaenoic (HDoHE), hydroxyoctadecatrienoic (HOTrE), hydroxyeicosatetraenoic (HETE), and hydroxyeicosapentaenoic (HEPE) acids [5]. Figure 3 provides examples of LOX-derived oxylipins, the enzymes involved in their biosynthesis, and their specific receptors. In humans, six lipoxygenases are distinguished: 15-LOX-1 (sometimes 12/15-LOX), 15-LOX-2, 12-LOX (platelet-type, pl12-LOX), 12R-LOX, eLOX3, and 5-LOX, encoded by the genes *ALOX15, ALOX15B, ALOX12, ALOX12B, ALOXE3*, and *ALOX5*, respectively [67]. As with COX-derived metabolites, LOX-derived oxylipins often mediate their effects by binding to G-protein coupled receptors, though specific targets have been identified for only a subset (Figure 3).

Metabolites synthesized by lipoxygenases can be produced by different enzymes in different cells. For example, 14-HDoHE, a metabolite of DHA, can be produced by 15-LOX in neutrophils [68] or by 12-LOX in platelets [69]. LOX-derived oxylipins can exert diverse and sometimes opposing effects. For instance, 4-HDoHE, 11-HDoHE, and 17-HDoHE are considered important pro-resolving mediators [70,71], while 5-HETE and 8-HDoHE possess pro-inflammatory properties [72,73].

Several oxylipins promote atherosclerotic processes through inflammatory mechanisms. 12(S)-HETE enhances monocyte-endothelial interactions, promotes smooth muscle cell proliferation, and impairs macrophage efferocytosis. Elevated 12-HETE levels have been observed in patients with coronary artery disease compared to healthy controls [74]. The study by [75] observed elevated levels of arachidonic acid and its metabolite, leukotriene B4, in atherosclerosis. Atherosclerotic aortas show increased 15-HETE synthesis, which exhibits chemotactic properties for smooth muscle cells and mitogenic effects on endothelial cells. 15-HETE also inhibits prostacyclin synthesis, potentially promoting thrombosis [76]. LTB4 promotes neutrophil recruitment, endothelial activation, and foam cell formation. Studies demonstrate increased LTB4 levels in atherosclerotic plaques and an association with plaque instability [77]. Conversely, in mouse models of atherosclerosis, genetic or pharmacological inhibition of 5-LO and FLAP effectively suppressed the formation and progression of atherosclerotic plaques [78]. This suggests that regulating leukotriene biosynthesis could be a novel anti-inflammatory strategy for the prevention and treatment of atherosclerotic cardiovascular diseases [78].

Inhibition of proteins in the lipoxygenase pathway is attracting attention, and a number of drugs have been developed. The 5-LOX inhibitor atreleuton, in a phase II clinical trial, reduced the progression of atheroma [79]; however, the treatment did not lead to a reduction in vascular inflammation [80]. The connection between leukotriene biosynthesis and atherosclerotic cardiovascular disease is discussed in more detail in the review [78]. The inhibitor of 5-lipoxygenase-activating protein (FLAP), AZD5718, demonstrated suppression of leukotriene biosynthesis in phase I [81,82]. A critical limitation is the blockade of both pro-inflammatory leukotrienes and anti-inflammatory lipoxins, but not DHA-derived SPMs [83].

An imbalance between pro- and anti-inflammatory mediators as a potential mechanism for the initiation and progression of atherosclerotic plaques is supported by recent studies. A study of serum levels of resolvin E1 (RvE1) and leukotriene B4 (LTB4) in 34 patients with coronary artery atherosclerotic plaques and 32 healthy controls [84] revealed a significant disruption in the balance between anti-inflammatory and pro-inflammatory mediators. Patients with atherosclerosis exhibit an imbalance between specialized anti-inflammatory mediators (pro-resolving) and pro-inflammatory factors. Despite a significant increase in both the pro-inflammatory leukotriene B4 (LTB4) and the anti-inflammatory RvE1 and its precursor EPA in the serum, the key indicator—the RvE1/LTB4 ratio—was sharply (more than 2 times) reduced. This imbalance may be a key reason for the transition from acute to chronic inflammation in atherosclerosis.

These findings are supported by the study [85], which investigated the association between specialized pro-resolving mediators (SPMs) and the progression of coronary plaques in 31 patients with stable coronary artery disease (CAD) receiving statin therapy. Coronary plaque volume was measured using coronary computed tomographic angiography at baseline and at 30-month follow-up. Patients were divided into a group receiving EPA+DHA and a control group. Measurements showed that a higher plasma EPA+DHA level was associated with a significant increase in the concentration of two SPMs (RvE1 and Mar1), as well as their precursor (18-HEPE). It was found that the key factor is the balance between pro- and anti-inflammatory mediators: a low value of the (18-HEPE + RvE1)/LTB4 ratio was associated with plaque progression; an increased (18-HEPE + RvE1)/LTB4 ratio led to plaque regression. The beneficial effect of omega-3 fatty acids in CAD appears to be mediated by the restoration of the balance between pro-resolving and pro-inflammatory mediators, which influences the dynamics of atherosclerotic plaques. The main challenge in applying SPMs in clinical practice is the rapid inactivation of natural SPMs. Metabolically stable analogs (e.g., lipoxin mimetics) [86,87,88] and targeted delivery systems [89] are being developed. Thus, therapeutic strategies aimed at restoring the SPM balance may represent a promising approach to stabilizing atherosclerotic plaques and preventing acute coronary events.

At this stage of research, our understanding of the potential mechanisms for redirecting PUFA metabolism from the synthesis of predominantly pro-inflammatory compounds to pro-resolving (inflammation-resolving) ones is relatively limited. Numerous mechanisms may be involved in shifting the normal response towards inflammation (low RvE1/LTB4 ratio) in the vascular wall and plaque instability [90,91]. These include increased activity of the 5-LOX enzyme and reduced availability of EPA (a substrate for the synthesis of resolvins RvE1, RvD1); involvement of additional signaling pathways (cytokines (TNF-α, IL-1β) and oxidized LDL enhance LTB4 production); suppression of factors promoting inflammation resolution (PPAR-γ, SIRT1); and reduced efficacy of pro-resolving mediators due to receptor dysfunction (e.g., ChemR23) and impairment of efferocytosis [2,90,92,93]. Oxidative stress also plays a role: in plaques, reactive oxygen species degrade pro-resolving mediators and exacerbate inflammation [94].

### 3.3. The Cytochrome P450 (CYP) Pathway

**Figure 4:**
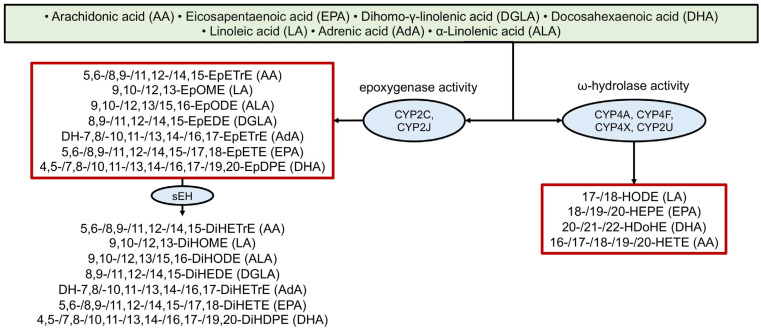
Biosynthesis of oxylipins via the cytochrome P450 (CYP) pathway. The ovals show the main enzymes of oxylipin biosynthesis, and the precursor acids are indicated in brackets. Abbreviations: Metabolites: EpETrEs—Epoxyeicosatrienoic acids, EpOMEs—epoxyoctadecamonoenoic acids, EpODEs—epoxy-octadecadienoic acids, EpETrEs—epoxyeicosatrienoic acids, EpETEs—epoxyeicosatetraenoic acids, EpDPEs—epoxyeicosapentaenoic acids, DiHOMEs—dihydroxyoctadecamonoenoic acids, DiHEDEs—Dihydroxy-eicosadienoic acids, DiHETrEs—dihydroxyeicosatrienoic acids, DiHETEs—dihydroxyeicosatetraenoic acids, DiHODEs—dihydroxyoctadecadienoic acids, DiHDPEs—dihydroxydocosapentaenoic acids, HODEs—hydroxyoctadecadienoic acids, HEPEs—hydroxyeicosapentaenoic acids, HDoHEs—hydroxydocosahexaenoic acids, HETEs—hydroxyeicosatetraenoic acids; Proteins: sEH—soluble epoxide hydrolase, CYP—cytochrome P450.

The oxidation of arachidonic acid by cytochrome P450 enzymes was described relatively early (see, for example, [95]). More recently, derivatives of other PUFAs have been analyzed, although a clear understanding of which specific enzymes are involved in their metabolism and their degree of specificity is still lacking [4,5]. Figure 4 provides examples of several CYP-derived oxylipins and the enzymes involved in their biosynthesis. CYP enzymes are actively involved in oxylipin biosynthesis, interacting with all major PUFAs and producing various metabolites (e.g., EpETrEs/HETEs from AA, EpOMEs from LA, EpODEs from ALA, DGLA-EpEDEs, DH-EpETrEs from AdA, EpETEs/HEPEs from EPA, EpDPEs/HdoHEs from DHA).

Many of these epoxy-metabolites are subsequently converted into more stable dihydroxy-compounds by soluble epoxide hydrolase (sEH) [96], yielding AA-DiHETrE, LA-DiHOME, ALA-DiHODE, DGLA-DiHEDE, AdA-DH-DiHETrE, EPA-DiHETE, and DHA-DiHDPE, respectively) (Figure 4).

Using AA as an example, at least 8 CYP enzymes are involved in the biosynthesis of EETs and HETEs [97], and some of them were proposed as a target for drug development to treat atherosclerosis and hypertension induced by hypercholesterolemia [98]. Two main groups are distinguished: CYP2J and CYP2C, which possess epoxygenase activity, and CYP4 families with ω-hydroxylase activity, which are major contributors to CYP-mediated PUFA derivatives [97].

Physiologically, EETs exert vasodilatory effects [99] and reduce apoptosis in rat cardiomyocytes after hypoxia and reoxygenation [100]. In contrast, some LA derivatives like certain HODEs are PPARγ agonists [101], while DiHOMEs can be cytotoxic and exhibit both pro- and anti-inflammatory properties [5]. Several hypotheses explain the actions of EETs at the cellular level. They can bind to nuclear PPAR receptors, primarily PPARγ, and regulate its activity [101]. The existence of a specific G-protein coupled receptor for EETs has been suggested, but functional screening has not yet identified a high-affinity receptor [102].

Epoxyeicosatrienoic acids (EpETrE, also abbreviated as EETs) exhibit multiple cardioprotective effects, including vasodilation, anti-inflammatory actions, and protection against ischemia–reperfusion injury. EET levels are inversely correlated with coronary artery disease severity. Genetic polymorphisms affecting EET metabolism are associated with increased cardiovascular risk [74,103]. CYP-derived oxylipins can limit the accumulation of inflammatory monocytes and promote repair [96].

sEH is a key modulator governing the balance between bioactive epoxy fatty acids and their generally less active dihydroxy derivatives. It was suggested as a defining marker of atheroprone endothelial cells and is further upregulated during atherosclerosis development [104]. The link between sEH activity and pathology has been previously indicated [105,106]. Epoxyeicosatrienoic acids (EETs) are cardioprotective, but they are inactivated by soluble epoxide hydrolase (sEH) [103,107]. Recently, it was shown that sEH elevation precedes plaque formation [104]. Studies demonstrate that sEH inhibition reduces atherosclerotic lesion formation in animal models; it reduces pro-inflammatory gene expression in endothelial cells, improves lipid profiles (decreased LDL, increased HDL), and enhances endothelial function [103,104]. In addition to regulating inflammation, sEH affects mitochondrial function (via mitochondrial complex I) and metabolic balance in endothelial cells [104]. This discovery, together with the known positive effects of sEH inhibitors, justifies the feasibility of targeted therapy. Specific suppression of sEH in the endothelium represents a new promising strategy for treating atherosclerosis. Preclinical data are promising; however, the clinical efficacy of sEH inhibitors (AR9281, GSK2256294) is limited [108]. Another line of research is focused on developing stable EET analogs [108,109,110].

It should be noted that not all derivatives of ω-3 fatty acids are anti-inflammatory. For instance, the DHA metabolite 19,20-DiHDPE, by inhibiting mitochondrial complex I, triggers oxidative stress leading to atherogenic and pro-inflammatory activation of the endothelium [104]. These important data indicate the possibility of switching between pro- and anti-inflammatory properties of metabolites in the epoxygenic transformation pathway of PUFA.

### 3.4. The Anandamide Pathway

The metabolites of the anandamide pathway are not classified as oxylipins; they are collectively referred to as N-acyl ethanolamines (NAEs) and 2-mono-acyl-glycerols (2-MAGs) [111,112]. The terms anandamide-like compounds or endocannabinoids (eCBs) are sometimes used, which also include compounds derived from saturated fatty acids, tetrahydrocannabinol, and other analogs that constitute the endocannabinoid system (ECS) [18]. The metabolites of the anandamide pathway are considered within a unified system with oxylipins because NAEs are formed from the same polyunsaturated fatty acids (PUFAs), in response to similar stimuli, and their metabolism involves phospholipases A2 and other enzymes from oxylipin synthesis pathways; the formation of these compounds depends on the PUFA content in the diet [111,113,114]. All this justifies not only the use of the term endocannabinoidome for this group of compounds [18] but also considering this group within a unified system with PUFA oxy-derivatives [114]. Like other PUFA derivatives, compounds in this group are actively involved in innate immune responses and possess both pro-inflammatory and anti-inflammatory properties (as referenced elsewhere in this text). Like oxylipins, NAEs are involved in the pathogenesis of atherosclerosis and other cardiovascular diseases [53,115].

The name “anandamide pathway” of PUFA metabolism is a tribute to tradition, as this pathway was historically most studied for the arachidonic acid (AA) derivatives anandamide (AEA) and 2-arachidonoylglycerol (2-AG); it is also part of the endocannabinoid system (ECS) [18,112]. The ECS consists of biosynthetic and degradative enzymes for eCBs and includes corresponding membrane receptors and other molecular targets [18,112]. It has been shown that the ECS plays a role in the development and progression of atherosclerosis [53,115]. Elevated levels of endocannabinoids are known to occur in atherosclerosis in both humans and experimental animals [116,117,118]. The role of this increase is ambiguous, as the mechanisms of synthesis and metabolism of these compounds in cells involved in atherogenesis are not fully understood.

Like oxylipins, AEA is involved in regulating the functions of all cells implicated in the pathogenesis of cardiovascular disease [116,119,120]. Using macrophage-derived foam cells and a cell metabolomics strategy, it was shown that the uptake of oxidized low-density lipoproteins (oxLDL) by macrophages disrupts the synthesis and metabolism of anandamide, leading to its accumulation and subsequent metabolic disturbances [119]. The authors demonstrated that arachidonic acid metabolism contributes to early atherosclerosis and proposed AEA as a biomarker capable of distinguishing foam cells from control cells [119]. Platelets can increase endocannabinoid synthesis during the formation of atherosclerotic plaques; in turn, AEA and 2-AG can activate human and rodent platelets, potentially contributing to prothrombotic effects [116]. AEA stimulates the release of NO from endothelial cells, promoting vasodilation and potentially exerting an anti-atherogenic effect [120]. AEA can bind to both specific G-protein-coupled receptors (cannabinoid receptors 1 (CB1) and 2 (CB2)) and other targets, such as the PPAR transcriptional factors [121]. Activation of CB1 receptors primarily exerts pro-inflammatory and pro-atherogenic effects (stimulation of monocyte adhesion, macrophage activation), whereas stimulation of CB2 receptors is associated with anti-inflammatory and anti-atherogenic effects (suppression of macrophage migration, induction of apoptosis) [115]. It is suggested that activation of CB2 receptors may represent an attractive therapeutic strategy, as, i.e., CB2 agonists reduce oxLDL accumulation in macrophages and modulate the production of inflammatory cytokines [122,123]. It was recently shown that AEA increases the expression of nuclear receptors NR4A1 and NR4A2 (not via CB1 or CB2), leading to suppressed expression levels of pro-inflammatory genes [124]. By binding to NR4A, AEA triggers an anti-inflammatory response in Human aortic smooth muscle cells (HAoSMC) [124]. AEA can interact with enzymes such as 5-/12-/15-LOX, transforming into hydroxyeicosatetraenoyl-ethanolamides, with COX-2, transforming into prostaglandin-ethanolamides, and via the CYP pathway, converting into epoxyeicosatrienoic acid-ethanolamides [114,125,126]. The role of NAEs derived from other PUFAs is not entirely clear. Studies on 3T3-L1 adipocytes have shown that they are indeed able to convert docosahexaenoic acid (DHA) and eicosapentaenoic acid (EPA) to their NAE derivatives [127]. It was demonstrated that these derivatives from ω-3 PUFAs possess anti-inflammatory properties in an LPS-stimulated model of inflammation. Docosahexaenoyl ethanolamine (DHEA) has also been found in human plasma [127]. This further emphasizes that the metabolites of the anandamide pathway of PUFA transformation should be considered in conjunction with other oxylipins.

The serum levels of AEA and other NAEs can be affected by the daily intake of ω-3 and ω-6 PUFAs [113]. Some effects of PUFA-enriched diets may be explained by the formation of corresponding NAEs and other ECS compounds. It is also known that anandamide (AEA) levels increase with physical exercise [114]. The positive role of moderate physical exercise in atherosclerosis has already been demonstrated [128,129], and increased synthesis of NAEs may be one of the underlying mechanisms. Besides dietary regulation, the impact of low-molecular-weight substances on the anandamide pathway is being studied. For instance, the discovered involvement of NAEs in inflammatory processes stimulates the search for inhibitors of their biosynthesis as potential anti-inflammatory agents [126]. The biosynthesis of AEA and other NAEs occurs from membrane phospholipids via various enzymatic pathways. The most studied of these is the pathway involving N-acyltransferases and N-acyl-phosphatidylethanolamine (NAPE)-specific phospholipase D [125]. Fatty acid amide hydrolase (FAAH) is one of the key enzymes responsible for the breakdown of AEA to AA. FAAH deficiency leads to enhanced recruitment of neutrophils into atherosclerotic plaques, accompanied by a local increase in the neutrophil chemoattractant CXCL1 in the aortas of FAAH-deficient mice, as well as an enhanced pro-inflammatory immune response [118].

Thus, the role of anandamide pathway metabolites in atherosclerosis is multifaceted and context dependent. Although, in general, as with oxylipins, derivatives of ω-3 acids exhibit anti-inflammatory properties, a more detailed analysis reveals the oversimplification of this approach. This is evident in the case of the well-studied anandamide. On one hand, anandamide exerts anti-inflammatory effects by suppressing the expression of adhesion molecules in endothelial cells, activating NR4A nuclear receptors, and modulating macrophage function via CB2 receptors. On the other hand, elevated levels of anandamide may promote neutrophil recruitment, platelet activation, and the formation of a more vulnerable atherosclerotic plaque phenotype.

### 3.5. Non-Enzymatic Oxidation

Oxylipins can be formed non-enzymatically through free radical-induced lipid peroxidation of PUFAs [54]. The role of non-enzymatic oxylipins in atherosclerosis began to be investigated as early as 1997, when the study [130] showed an increased content of F2-IsoPs (8-epi-PGF2α and IPF2α-I) in human atherosclerotic plaques compared to control samples. Currently, one of the most studied classes of oxylipins formed under conditions of stress are isoprostanes, derivatives of arachidonic, eicosapentaenoic, docosahexaenoic, and adrenic acids, designated as F2-IsoPs, F3-isoprostanes (F3-IsoPs), F4-neuroprostones (F4-NeuroPs), and F2-dihomo-isoprostanes (F2-dihomo-IsoPs), respectively. A number of LA derivatives, such as 9-HODE and 13-HODE, can also be synthesized non-enzymatically [131].

Products of non-enzymatic PUFA oxidation are often considered markers of oxidative stress in various diseases, including cardiovascular diseases [54,132]. In vivo studies have shown that impairments in the cardiovascular system are associated with an increased level of F2-IsoPs (8-F2t-IsoP, 5-F2c-IsoP and 5-epi-5-F2t-IsoP) and a decreased level of F4-NeuroPs (10-F4t-NeuroP, 20-F4t-NeuroP) in model mice (deficient in very long-chain acyl-CoA dehydrogenase (VLCAD)) compared to controls [133]. Furthermore, when DHA was added to the diet of atherosclerosis-susceptible mice (LDL receptor knockout mice), the hepatic content of F4-neuroprostones negatively correlated with plaque prevalence [134].

F2-isoprostanes, formed from arachidonic acid, are reliable biomarkers of oxidative stress in human cardiovascular diseases [135]. Their plasma levels correlate with the severity of coronary atherosclerosis and the number of affected vessels. A threshold value of 124.5 pg/mL (measured using a commercial EILSA kit from Cayman Chemical) has 74% sensitivity and 81% specificity for predicting cardiac events, surpassing the predictive ability of high-sensitivity C-reactive protein. This is supported by data showing that in patients with acute coronary syndrome, isoprostane levels are more than 3 times higher than in patients without ACS, with 42% of patients in the upper tertile reaching primary endpoints within 30 days [136]. Thus, non-enzymatically oxidized oxylipins, particularly F2-isoprostanes and F4-neuroprostones, can be proposed as promising biomarkers for cardiovascular diseases associated with oxidative stress. In summary, it can be said that while non-enzymatic oxidation is a significant mechanism for converting LDL into atherogenic particles and for direct tissue damage, and although some oxylipins can be formed via both pathways, it is the enzymatic pathways that drive the cellular response. Clinical trials of antioxidants aimed at suppressing oxidative stress, which have demonstrated the failure of this strategy, also support the effectiveness of pursuing the possibility of modulating cellular responses rather than directly suppressing non-enzymatic reactions [94].

### 3.6. Challenges in the Oxylipins System Investigations

Analyzing the oxylipin metabolism system leads to several conclusions: (1) PUFAs and their oxylipin derivatives are important signaling molecules involved in numerous diseases associated with disturbance of the innate immune system and chronic inflammation; (2) upon cell activation, a multitude of diverse oxylipins, often with opposing actions, are formed simultaneously; (3) oxylipins act through various receptors, meaning different signaling pathways are activated concurrently.

For many decades, research focused on characterizing individual signaling lipids. However, the processes of the initiation, development, and chronicity of endogenous inflammation remain insufficiently understood to be effectively managed. It is now evident that new approaches to studying these classes of substances are needed. Signaling lipids likely emerged with the first cells. They are found even in unicellular eukaryotes, such as yeast and ciliates [18]. This is not surprising, given that signaling lipids are part of the innate immune system, whose origins are also traced back to unicellular organisms [137,138]. Perhaps comparing oxylipin profiles across different organisms will enable the development of new systems for managing PUFA metabolism and the effects of signaling lipids.

The complexity of the biological system of PUFA metabolism makes it difficult to transfer in vitro and in vivo data to humans. Enzymes that metabolize polyunsaturated fatty acids into oxylipins—such as cyclooxygenases (COX), lipoxygenases (LOX), and cytochrome P450 (CYP)—exhibit species-specific differences in their expression and activity. For instance, rodents and humans have different CYP isoforms and activities, leading to the formation of distinct oxylipin profiles, such as epoxidized products and hydroxyeicosatetraenoic acids (HETEs) [139]. Furthermore, genetic and epigenetic variations affecting the expression of these enzymes can differ significantly between species. Studies show that genetic factors are a major contributor to the variability of oxylipins synthesized by 12-LOX (e.g., 12-HETE and 12-HEPE), and these influences may not be consistent between animals and humans [140].

The concentration and spectrum of oxylipins can vary significantly in the same tissues across different species, affecting local signaling and function. Adipose tissue serves as a clear example. While in mice the total oxylipin content is lower in brown adipose tissue (BAT) compared to white adipose tissue (WAT), the opposite pattern is observed in humans: oxylipin levels are higher in BAT than in WAT [141]. These differences are primarily associated with variations in the levels of oxylipins derived from linoleic acid (9- and 13-HODE). Although the total amount of PUFA metabolites in tissues may be similar between mice and humans, the distribution among specific classes of oxylipins and endocannabinoids differs, indicating species-specific characteristics in precursor pool distribution [141].

Thus, it can be noted that species differences in the metabolism and functions of oxylipins, including variations in enzyme expression, tissue-specific profiles, and the balance of pro-inflammatory and pro-resolving signals, can significantly impact the interpretation of experimental data. This necessitates caution when extrapolating results obtained from animal models to humans. To overcome these translational challenges, rigorous validation of any promising oxylipin biomarkers and therapeutic targets in large-scale clinical studies is required.

## 4. Oxylipin Profiles as Biomarkers of Atherosclerosis: Diagnostic and Prognostic Potential

Although individual oxylipins are released at relatively low concentrations, their effects can be additive from the sum of similar influences, which can potentially be managed [142,143]. This points to the promise of seeking ways to manage oxylipin profiles rather than individual metabolites.

These properties impose special requirements for their study, primarily the acquisition and analysis of data using multi-omics methods, and first and foremost, metabolomic analysis of the oxylipin profile.

Studying oxylipin profiles in various diseases demonstrates their diagnostic and prognostic potential [8]. In rheumatoid arthritis, oxylipin profiling helped identify clinically significant patient clusters and predict treatment response [9]. Links between oxylipin profiles and type 2 diabetes have been established [10]. Cancer research has revealed specific oxylipin patterns in patients with various malignancies [see review [11]]. Oxylipins are being investigated as biomarkers in various ophthalmological and neurological diseases [12,13,14].

The observation that the plasma oxylipin spectrum changes with disease progression in Parkinson’s disease is also interesting [144]. Furthermore, the oxylipin profile not only reflects disease stages but can also reveal new therapeutic targets [144]. In patients with early-stage Parkinson’s (Hoehn and Yahr stages I-II), a weakened contribution of oxylipins to immune homeostasis was observed, while in advanced disease (stages III-IV), increased synthesis of pro-inflammatory metabolites from the CYP (19-HETE), COX (PGD2, PGA2, PGJ2) and LOX (9-KODE, 13-HODE, 8-HDoHE, 12-HETE, 13-KODE) pathways was found [144]. It has been proposed to add inhibitors of these enzymes to standard therapy for specific cohorts of patients with advanced disease. The prospects of using oxylipin supplements, such as anandamide or SPMs, in the early and middle stages are also discussed [144].

Modern clinical studies demonstrate the significant diagnostic potential of oxylipin profiling in coronary artery disease. The study by Huang et al. [145] included 2239 patients with stable CAD. Among them, 25 who experienced an acute myocardial infarction (AMI) within two years were examined, along with a control group of 50 matched patients without events. A comprehensive analysis of 46 oxylipins and PUFAs was conducted. Kaplan–Meier analysis showed that future AMI was more frequent in patients with higher baseline levels of arachidonic acid derivatives: pro-inflammatory LOX derivatives 8-HETE, 9-HETE, 11-HETE, 12-HETE, 15-HETE, as well as vasoactive CYP derivatives epoxyeicosatrienoic acids 5,6-EET, 8,9-EET, 11,12-EET, 14,15-EET and 19-HETE, 20-HETE, suggesting that a general disruption in arachidonic acid metabolism, which shifts production toward these specific oxylipins by activating these LOX/CYP enzymatic pathways, serves as a significant prognostic biomarker for cardiovascular risk. Multivariate Cox analysis confirmed that patients with high baseline levels of these oxylipins had a significantly increased risk of AMI [145].

The study by Le et al. [74] assessed the diagnostic and prognostic potential of plasma oxylipins in coronary heart disease. The study included 74 participants with varying numbers of affected coronary arteries and 23 low-risk participants. A targeted analysis of 39 oxylipins was performed. The plasma levels of six oxylipins decreased with an increasing number of affected arteries (12,13-DiHOME—a LA derivative; LTB4—an AA derivative; 8-iso PGF3α—an EPA derivative via the ROS pathway; and DHA derivatives 19,20-DiHDPA, 13,14-DiHDPA and 16,17-DiHDPA). A panel of five oxylipins (DHA derivatives formed via the CYP pathway—19,20-EpDPA, 13,14-DiHDPA; 10,11-DiHDPA and 19,20-DiHDPA (under the action of sEH) and LTB4—an AA derivative formed via the 5-LOX pathway) allowed for the detection of three affected arteries with 100% sensitivity and 70% specificity. For predicting 5-year survival, a panel of two oxylipins (9-HODE, a LOX-derived LA metabolite and 10,11-EpDPA, a CYP-derived DHA metabolite) was proposed, with a sensitivity of 86% and a specificity of 91% [74].

## 5. Challenges in the Clinical Analysis of Oxylipins

Modern platforms allow for the simultaneous quantitative determination of hundreds of oxylipins, representing significant progress compared to early immunoassay methods which could only analyze single compounds [146,147].

In modern approaches to oxylipin analysis, the most common system combines reversed-phase chromatography with ultra-high performance liquid chromatography (UHPLC). The use of sub-2 µm particles in UHPLC provides high resolution, speed, and sensitivity while reducing analysis time and solvent consumption. Detection is performed using triple quadrupole (QqQ) mass spectrometry, where electrospray ionization (ESI) in negative mode and multiple reaction monitoring (MRM) are the standards. This ensures high specificity and sensitivity for the detection and differentiation of structurally similar oxylipins [146,147]. Despite the significant potential of oxylipins as biomarkers for inflammatory and metabolic diseases, their translation from research to clinical diagnostics remains limited. The main obstacles are the lack of standardized analytical protocols, high analysis costs, and the complexity of clinical validation. The study of oxylipins has long been challenging, primarily due to the technical difficulty of detecting these lipid compounds, whose concentration in biological fluids is only 1–100 pmol/liter [148].

To date, over 200 molecules from the oxylipin group exhibiting physiological activity have been discovered [5]. Notably, this represents a significant portion of the total lipid content in blood, where just over 600 lipids have been identified in total [149]. Standard conditions for measuring the lipid profile for use in clinical practice have been established [150].

### 5.1. Core Problem: Lack of Method Standardization and Harmonization

To become a clinically significant biomarker, an oxylipin must undergo three stages: discovery, analytical validation, and clinical validation. No oxylipin has undergone full clinical validation, which requires large-scale, multi-center studies using standardized protocols [8,19]. Concentrations of the same oxylipin reported in different studies can vary by orders of magnitude. This is due to both biological variability and differences in analytical methods. Efforts are underway to harmonize protocols [148]. Studies have shown that when using unified protocols, common materials, and standard calibrations, the technical error for 73% of oxylipins can be reduced to within ±15% [151]. This proves that obtaining reproducible results between laboratories is achievable.

### 5.2. Critically Important Preanalytical Factors

Oxylipins are highly unstable during sample collection, processing, and storage. Strict adherence to protocols at this stage is a mandatory requirement for obtaining reliable data. EDTA-plasma is the preferred matrix for oxylipin analysis. Serum formation is associated with a significant increase (by 1–3 orders of magnitude) in cyclooxygenase and 12-lipoxygenase products due to platelet activation during coagulation, with high inter-individual variability. This ex vivo formation makes it difficult to interpret serum concentrations as true circulating blood levels [19,152]. The stability of oxylipins depends on their biosynthetic pathway. Free cytochrome P450 and 5-lipoxygenase oxylipins, as well as autoxidation products, are minimally affected by storing whole blood for up to 4 h at 4 °C. Furthermore, the total amount of oxylipins (free and esterified) remains stable for up to 24 h at 4 °C and after automated transport. In contrast, COX products such as thromboxane B_2_ and 12-HHT, as well as products of 12-LOX enzymatic activity, are susceptible to changes induced by storage and transport due to platelet activation [152]. Samples must be kept on ice throughout collection and transfer, promptly centrifuged at 3000–4000 rpm for 5 min at 4 °C in a refrigerated centrifuge, and plasma should be immediately aliquoted into tubes and stored at −80 °C. Protecting samples from light throughout the process is necessary to prevent photodegradation. The use of antioxidants such as butylated hydroxytoluene (BHT) and EDTA in extraction buffers is critical to prevent autoxidation [153,154].

### 5.3. Analytical Challenges and Technical Limitations

The quantification of oxylipins faces a number of technical difficulties. Oxylipins are present in biological samples at very low concentrations (in the picomolar and nanomolar range), with a vast concentration difference exceeding 1000-fold between the least and most abundant species within a single sample. This requires analytical methods with both exceptional sensitivity and a wide linear dynamic range (ideally >3 orders) [19,147]. Many oxylipins are isomers (e.g., various HETEs and EETs) with identical mass spectra, requiring high-quality chromatographic separation for their differentiation [147]. It is impractical to use an individual deuterated standard for each of the 100–150 oxylipins (this would increase the cost of the analysis by orders of magnitude). The current practice (using ~11–37 standards, one per class) leads to matrix-dependent quantification errors. Expanding the range of labeled standards would significantly improve the accuracy and precision of the assay [147,154,155]. While the standard reference material NIST 1950 (Metabolites in Frozen Human Plasma) is available and characterized for 20 certified metabolite values overlapping with oxylipin panels, comprehensive certified reference materials specifically designed for the entire spectrum of oxylipins do not exist. This limits the ability to verify the accuracy of results across different laboratories and validate oxylipin detection methods [8,155].

### 5.4. Economic Barriers and Clinical Utility

The economic cost of oxylipin profiling is a significant obstacle to widespread clinical adoption. Cost Factors: Current prices for comprehensive oxylipin/eicosanoid panel analysis range from approximately $157 per sample for academic institutions to $253 per sample for external users in core facilities. These costs reflect expensive equipment (liquid chromatography coupled with tandem mass spectrometry, LC-MS/MS), the need for highly qualified personnel, complex sample preparation procedures, and the use of multiple deuterated internal standards [8,147]. Analysis of each sample takes between 15 to 30 min, depending on the complexity of the chromatographic method. Sample preparation is laborious and involves solid-phase extraction or protein precipitation, addition of internal standard mixtures, and often requires specialized equipment, such as automated extraction systems. To reduce the cost per sample and increase throughput, high-throughput approaches using 96-well plates have been developed, allowing for standardized processing of 50 µL plasma or serum samples [155]. However, even with these advances, the cost remains substantially higher than that of traditional clinical chemistry methods. Cost-Effectiveness and Alternative Assays: For clinical application, a cost-effectiveness analysis must demonstrate that oxylipin profiling provides sufficient diagnostic or prognostic value to justify these costs compared to existing biomarkers. Developing more targeted oxylipin panels specific to a particular disease, rather than comprehensive profiling, could reduce costs while retaining clinical value [8,19]. The availability of ELISA kits for detecting key oxylipin markers (3–4 compounds) could potentially offer an acceptable cost-effectiveness ratio, moving away from the use of expensive methods.

## 6. Conclusions and Future Directions

The traditional view of atherosclerosis as a disease of cholesterol accumulation has been fundamentally transformed by the recognition of its chronic inflammatory nature. This review has synthesized evidence demonstrating that oxylipins—a diverse family of bioactive lipids derived from polyunsaturated fatty acids (PUFAs) play a central role in the initiation, progression, and potential resolution of atherosclerotic inflammation.

The inflammatory response, essential for host defense, can become pathogenic when the delicate balance between pro-inflammatory and pro-resolving mediators is disrupted. Research on the evolution of PUFA metabolism in different organisms can provide academic insight into the principles of lipid signaling, which, in our view, is still far from being understood.

Although significant breakthroughs in understanding inflammation have occurred over the past two decades, a complete understanding is still lacking. It is a context-dependent and evolving process, in which the dual role of oxylipins must be a focus of research. Metabolites from the COX and 5-LOX pathways (e.g., PGE2, LTB4, 12-HETE) predominantly contribute to endothelial dysfunction, leukocyte recruitment, and plaque instability. In contrast, specialized pro-resolving mediators (SPMs) such as resolvins and lipoxins, along with anti-inflammatory epoxyeicosatrienoic acids (EETs), actively promote inflammation resolution, tissue repair, and plaque stabilization. The critical importance of this balance is underscored by clinical studies showing that the ratio of pro-resolving to pro-inflammatory oxylipins (e.g., RvE1/LTB4) is a more powerful indicator of plaque regression or progression than the absolute levels of individual mediators. It is necessary to investigate oxylipin profiles and their changes in response to anti-inflammatory agents at various stages of atherosclerosis development.

Advancements in mass spectrometry-based lipidomics have been pivotal, enabling the simultaneous quantification of hundreds of oxylipins and moving the field beyond the study of single molecules. This comprehensive profiling has revealed distinct oxylipin signatures associated with the severity of coronary artery disease, the risk of future myocardial infarction, and patient survival. These findings highlight the tremendous potential of oxylipin panels as diagnostic and prognostic biomarkers, capable of identifying high-risk patients long before clinical events occur. The future direction involves building comprehensive metabolomic datasets for oxylipins. Establishing a unified database of these results, akin to those in transcriptomics, would greatly accelerate progress in this field.

From a therapeutic perspective, targeting oxylipin pathways presents promising avenues beyond traditional approaches. While approved anti-inflammatory drugs such as colchicine and canakinumab represent significant steps forward, they target downstream effectors of inflammation. Interventions aimed at directly modulating oxylipin networks—such as stimulating SPM synthesis through omega-3 supplementation, inhibiting the 5-LOX/LTB4 axis, or preserving cardioprotective EETs via soluble epoxide hydrolase (sEH) inhibition—hold the potential for more precisely restoring the balance between inflammation and resolution. Furthermore, the complex role of endocannabinoids like anandamide illustrates the context-dependent nature of these lipids, suggesting that selective modulation of specific receptors (e.g., CB2) may yield beneficial effects. We believe the future of this field belongs to low-molecular-weight compounds developed not merely to inhibit the synthesis of a particular oxylipin or one PUFA metabolic pathway but rather designed with consideration for the alterations in polyenzymatic cascades upon the blockade of a single site. This will lead to greater efficacy and fewer side effects.

In conclusion, oxylipin metabolism represents a critical link between dietary lipids, innate immunity, and vascular pathology in atherosclerosis. Future research should focus on longitudinal studies to validate specific oxylipin panels as clinical biomarkers and on developing targeted therapies that shift the balance from chronic inflammation to active resolution. Furthermore, academic research using cell lines and animal models should elucidate the physiological activities of oxylipin profiles, their correlation with pathology development stages, and the possibilities for correction not just by targeting a single metabolic branch, but through modulating different branches of oxylipin metabolism and signaling. Harnessing the potential of the oxylipin system promises to open new frontiers in the personalized prediction, prevention, and treatment of atherosclerotic cardiovascular diseases.

## Data Availability

No new data were created or analyzed in this study. Data sharing is not applicable to this article.
